# Preoperative white blood cell count predicts anastomotic leakage in patients with left-sided colorectal cancer

**DOI:** 10.1371/journal.pone.0258713

**Published:** 2021-10-20

**Authors:** Masaki Morimoto, Kenjiro Taniguchi, Osamu Yamamoto, Takuji Naka, Atsushi Sugitani, Yoshiyuki Fujiwara

**Affiliations:** 1 National Hospital Organization, Yonago Medical Center, Yonago, Japan; 2 Faculty of Medicine, Division of Gastrointestinal Surgery, Department of Surgery, Tottori University, Yonago, Japan; Osaka Medical Center for Cancer and Cardiovascular Diseases, JAPAN

## Abstract

To determine whether preoperative white blood cell (WBC) counts reflect risk of anastomotic leak (AL) for patients with colorectal cancer (CRC), we retrospectively examined data from records of 208 consecutive patients who had undergone resections for left-sided CRC, including their clinicopathological parameters and preoperative laboratory data. The diagnostic value of WBC count for AL was evaluated and compared with those of neutrophil-lymphocyte ratio, platelet-lymphocyte ratio, lymphocyte-monocyte ratio and platelet count × C-reactive protein level multiplier (P-CRP) value; optimal cut-off values were derived from receiver operating characteristic curves. AL was observed in 11 of the 208 patients (5.3%). Compared with the no-AL group, the AL group had a significantly higher mean WBC count and smoking rate. In multivariate analysis, WBC count and smoking were independent risk factors for AL. Compared with the other tested inflammatory indicators, the cut-off value for WBC (6,200/μL) had the highest sensitivity (81.8%) and negative predictive value (98.4%), as well as the lowest likelihood ratio (0.289). Preoperative WBC count could therefore be a convenient predictor of AL in patients with left-sided CRC.

## Introduction

Colorectal cancer (CRC) is the third most commonly diagnosed cancer in men and the second-most common cancer in women [1]. A serious complication of surgery for CRC is anastomotic leakage (AL), a major cause of postoperative mortality and morbidity. Its incidence is reportedly 3%–30% [2,3]. AL not only causes mortality itself, but also delays adjuvant chemotherapy, thus increasing the risk of recurrence and fatal outcome. AL also increases the length and cost of hospitalization. For these reasons, AL prevention is a major consideration in colorectal surgery for patients with CRC.

Although some preoperative risk factors of AL for colorectal surgery have been suggested, no consensus has yet formed. No reliable detailed data about the relationship between AL and preoperative inflammation is available, especially for patients with left-sided CRC. In this study, we focused on the potential of preoperative inflammation-related indicators as risk factors for AL after surgeries for left-sided CRC.

## Materials and methods

### Patients

This retrospective study included 208 consecutive patients with left-sided (descending colon, sigmoid colon, rectum and anal canal) CRC who underwent elective colorectal resection and anastomosis at the National Hospital Organization, Yonago Medical Center (Yonago, Japan) between August 2014 and August 2020, either by open laparotomy or laparoscopic surgery. The location of left-sided CRC was determined by the Japanese Classification of Colorectal, Appendiceal, and Anal Carcinoma [4]. CRC patients were classified by clinical symptoms and pathological detection by the 8th edition of the Union for International Cancer Control TNM staging system.

Preoperative antibiotics were not used routinely and mechanical preparation of the colon was conducted by the individual physicians, typically using laxatives for 1–2 day(s) before operation. All patients received postoperative antibiotic prophylaxis. Prophylactic antibiotics (e.g., flomoxef) were administered within 30 min before skin incision, and then every 3 h intraoperatively. Prophylactic postoperative antibiotics were typically used until POD 1, and were the same antibiotics as those used intraoperatively.

The ethical review board of the National Hospital Organization, Yonago Medical Center, approved of the study (approval No. 0211–02) and the informed consent requirement was waived.

### Parameters

Patients’ clinicopathological and laboratory data were extracted from their electronic medical records, and included characteristics such as age, sex, body mass index (BMI), smoking, diabetes, neoadjuvant chemotherapy (NAC), tumor location, T/N stage, preoperative laboratory data, and surgical parameters such as operative approach, diverting stoma, anastomotic method, procedure duration and blood loss volume.

Data on preoperative serum C-reactive protein (CRP), white blood cell (WBC) count, cell counts for neutrophils, lymphocytes, monocytes, eosinophils, and platelets, and serum albumin levels, were collected within 1 month before their surgeries.

### Diagnosis of AL

AL was diagnosed through clinical and radiologic findings: (a) presence of air or abscess near the site of anastomosis detected on computed tomography (CT); (b) purulent or enteric discharge through the drainage tube; and/or (c) clinical signs of peritonitis and/or presence of fecal or purulent discharge during re-operation.

### Statistical analysis

Continuous variables are reported as means and standard deviations. Univariate analyses were performed using Fisher’s exact test for categorical variables and the Mann–Whitney *U*-test for continuous variables. Logistic regression analysis was used to calculate odds ratios (ORs) for multivariate analysis. Receiver operating characteristic (ROC) analyses were used to evaluate predictors for AL and to determine cut-off values. The cut off value was determined according to the point on the curve with minimum distance from the left-upper corner of the unit square, in order to compare the predicting properties between inflammatory factors. All statistical analyses were performed using the statistical software SPSS v. 23.0 statistical software (IBM Corporation, Armonk, New York, USA). *P* < 0.05 was considered significant.

## Results

In the period of this study, 208 patients underwent colorectal resection for left-sided CRC; all these patients were included in this analysis. Their median age was 69 (±11.1) years; 112 patients (53.9%) were men. Sigmoid colon cancer (*n* = 100, 48.1%) was the most common surgical indication, followed by rectosigmoid (*n* = 59, 28.4%), descending (*n* = 19, 9.1%), upper rectum (*n* = 18, 8.7%), lower rectum (*n* = 11, 5.3%) and anal canal (*n* = 1, 0.5%). The operative approaches were laparoscopic (*n* = 125, 60.1%) and open laparotomy (*n* = 83, 39.9%). Anastomoses were performed using a mechanical stapler in 122 patients (58.7%) and hand sutures in 86 patients (41.4%).

AL was observed in 11 of 208 (5.3%) patients; this cohort was defined as the AL group. Their AL was diagnosed between post-operative days (PODs) 1 and 13 (median: POD 4). To treat AL, 7 patients received ileostomies (64%), 3 received conventional drainage tubes (27%), and 1 underwent Hartmann’s operation (9%). The AL group suffered no AL-related deaths. The remaining 197 patients were classified as the no-AL group. Table 1 summarizes patient characteristics for the two groups. Mean age, sex, BMI, diabetes, neoadjuvant chemotherapy, pathological T and N statuses, tumor location, operative approaches, diverting stoma, anastomotic method, operation time, blood loss volume, preoperative CRP, preoperative proportions of neutrophils, lymphocytes, monocytes and eosinophils, preoperative platelets and preoperative albumin did not significantly differ between the AL and no-AL groups. However, the AL group had a significantly higher mean WBC (7000±2200/μL vs 5600±1700 /μL, *P* = 0.0023) and smoking rate (63.6% vs 18.3%, *P* = 0.0018).

**Table 1:** Associations between AL and clinicopathological factors.

		AL		
		Yes	No	
parameter		n = 11	n = 197	P value
Age (years)		67±11.2	69±11	0.226
Sex	Male	8	104	0.23
	Female	3	93	
BMI		21	22.2	0.517
Smoking	Yes	7	36	0.0018*
	No	4	161	
Diabetes	Yes	0	23	0.615
	No	11	174	
Neoadjuvant chemotherapy	Yes	0	13	1
	No	11	184	
pathological T	1/2	3	55	1
	3/4	8	142	
pathological N	negative	8	118	0.533
	positive	3	79	
Location	D/S/RS	8	171	0.185
	Ra/Rb/P	3	26	
Approach	open	5	78	0.757
	laparoscope	6	119	
Diverting stoma	Yes	1	37	0.693
	No	10	160	
Stenosis	Yes	1	32	1
	No	10	165	
Anastomotic method	hand sawn	2	84	0.128
	stapler	9	113	
Operation time (min)		242±62	204±83	0.241
Blood loss (g)		65±168	30±296	0.475
CRP (mg / dL)		0.35±1.1	0.11±2.1	0.157
White blood cell (10^3 /μL)		7±2.2	5.6±1.7	0.0023*
Neutrophil (%)		67±7.5	62±10.8	0.223
Lymphocyte (%)		20±8.7	27±8.8	0.0801
Monocyte (%)		5.9±1.9	6.3±2.3	0.435
Eosinocyte (%)		3.1±1.6	2.8±3.5	0.843
Platelet (10^4 /μL)		25.2±6.5	23.2±7.9	0.361
Albumin (g/dL)		4.1±0.51	3.9±0.55	0.737

body mass index; BMI, C-reactive protein; CRP, descending colon; D,sigmoid colon; S, rectosigmoid; RS, upper rectum; Ra, lower rectum; Rb, anal canal; P,

statistically significant; *.

In multivariate analysis, WBC count (OR: 1.51, *P* = 0.00059) and smoking (OR: 6.14, *P* = 0.00077) were independent risk factors for AL (Table 2).

**Table 2:** Multivariate analysis of prognostic factors for AL.

	OR	95% CI	P value
Smoking	6.14	1.31―23.4	0.0077*
White blood cell (10^3 /μL)	1.51	1.13―2.03	0.0059*

anastomotic leak; AL, odds ratio; OR, confidence interval; CI.

statistically significant; *.

**Fig 1:**
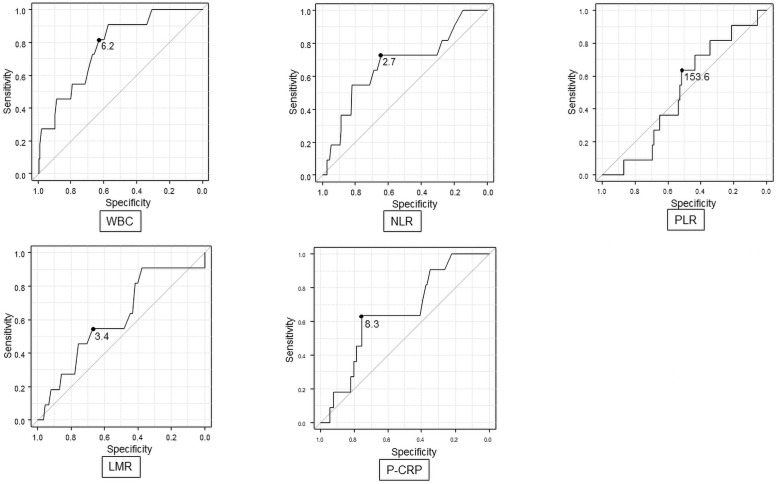
Receiver operating characteristic (ROC) curves and areas under the curve (AUC) of the possible predictors of anastomotic leakage. White blood cell; WBC, Neutrophil-lymphocyte ratio; NLR, Platelet-lymphocyte ratio; PLR, Lymphocyte-monocyte ratio; LMR, Platelet count × C-reactive protein level multiplier; P-CRP.

In consideration of the role that preoperative inflammatory indicators, such WBC count, play in diagnosing AL, we then examined WBC and other inflammatory indexes, including neutrophil-lymphocyte ratio (NLR) [5], platelet-lymphocyte ratio (PLR) [6], lymphocyte-monocyte ratio (LMR) [7] and platelet count × C-reactive protein level multiplier (P-CRP) value [8] with respect to AL, using ROC analyses. The cut-off values were WBC: 6,200/μL (area under the curve [AUC] = 0.773), NLR: 2.7 (AUC = 0.53), PLR: 153.6, (AUC = 0.503), LMR: 3.4 (AUC = 0.606) and P-CRP: 8.3 (AUC = 0.653; Table 3; Fig 1).

**Table 3:** AUC and cut-off values for clinical predictors of AL.

	AUC	95% C.I.	cut off
WBC (10^3 /μL)	0.773	0.644–0.902	6.2
NLR	0.673	0.49–0.855	2.7
PLR	0.503	0.357–0.649	153.6
LMR	0.606	0.429–0.784	3.4
P-CRP	0.653	0.498–0.808	8.3

area under the curve; AUC, anastomotic leak; AL, confidence interval; CI white blood cell; WBC, neutrophil-lymphocyte ratio; NLR, platelet-lymphocyte ratio; PLR, lymphocyte-monocyte ratio; LMR platelet count × C-reactive protein level multiplier value; P-CRP.

Finally, we used the cut-off values to determine the effectiveness of these indicators for AL. We found that WBC count was the most accurate, at 81.8% sensitivity, 98.4% negative predictive value, and 0.289 of negative likelihood ratio for predicting AL in our study cohort (Table 4).

**Table 4:** Diagnostic properties for clinical predictors of AL.

	Sensitivity	Specificity	PPV	NPV	positive LR	negative LR
WBC	0.818	0.629	0.11	0.984	2.208	0.289
NLR	0.636	0.65	0.092	0.97	1.817	0.56
PLR	0.455	0.482	0.047	0.941	0.878	1.131
LMR	0.455	0.31	0.035	0.91	0.658	1.762
P-CRP	0.545	0.756	0.111	0.968	2.239	0.601

anastomotic leak; AL, positive predictive value; PPV, negative predictive value; NPV likelihood ratio; LR, white blood cell; WBC, neutrophil-lymphocyte ratio; NLR, platelet-lymphocyte ratio; PLR, lymphocyte-monocyte ratio; LMR platelet count × C-reactive protein level multiplier value; P-CRP.

## Discussion

Patients with AL can experience fever, abscess, septicemia, metabolic disturbances, or multiple organ failure [9], which could lead to the need for reoperation, higher risk of local recurrence, increased morbidity and mortality and diminished general quality of life [10,11]. Therefore, various surgical techniques and prevention methods have been developed to overcome AL [12]. For example, recent prospective and retrospective studies show that the use of a trans- anal drainage tube significantly reduces AL [13,14] by lowering endo-luminal pressure at the anastomotic line.

To prevent the worst outcomes in patients with AL, early diagnosis, from indications and symptoms such as fever and peritonitis, is crucial. In recent years, CT, abdominal drain secretion analysis and biomarkers are the most commonly used strategies to diagnose AL [15–17].

If the patient’s ability to naturally heal is compromised, AL can occur, even if the surgery is without fault. Therefore, the ability to predict which patients are at high risk for AL would facilitate more careful monitoring and faster diagnosis for AL among these patients. Smoking [18], obesity [19] and male sex [20] are reported to be preoperative risk factors for AL. However, the current study found no significant differences in AL rates related to BMI and sex.

Thus, consensus on the role of preoperative factors for predicting AL risk in patients with CRC is lacking. Our study therefore focused on this unsolved issue, with a particular focus on inflammatory indicators’ predictive value for AL, using well-recorded preoperative data for our cohort.

The relationship between cancer and inflammation has been known ever since Rudolf Virchow first reported the presence of leukocytes within tumors in the 19th century [21]; the underlying molecular mechanisms are still obscure [22]. The contribution of inflammation and the immune system to cancer progression has driven a great deal of research. Several indicators based on common inflammatory factors, such as CRP, platelets and WBC, have prognostic value in various cancers [6,23,24]. These indicators have the advantages of simplicity and convenience, but the mechanisms by which they affect tumorigenesis are unclear. Nonetheless, a common mechanism underlying their prognostic value is an association with systemic and/or local inflammation.

Messias et al. reported that postoperative serum CRP levels in patients who undergo colorectal surgery could become a marker for the exclusion of anastomotic leakage [25]. Additionally, Smith et al. reported changes in CRP, WBC count and procalcitonin as potential markers of AL following colorectal surgery [26]. Those reports indicate that the postoperative trend of inflammatory indicators reflects the occurrence of AL. Actually, in this study, CRP and WBC at POD 3 were significantly higher in the AL group compared with the no-AL group (data not shown).

However, preoperative predictors for AL have not been widely studied. Although patients with cancer often have some inflammation that reflect their cancer progression, whether preoperative inflammatory status reflects AL risk in patients with CRC has not been examined.

To address this issue, this study analyzed preoperative predictors of AL in 208 patients who underwent resections for left-sided CRC. First, univariate analyses showed that the patients with AL had a significantly higher mean WBC count than did those without AL. Second, multivariate analysis showed that WBC count was independently related to AL risk. Third, ROC analyses showed that WBC count had the highest AUC (0.773), compared with NLR, PLR, LMR and P-CRP. Finally, high WBC count had the highest sensitivity and negative predictive value, and the lowest likelihood ratio, using the cut-off value (6,200/ μL). Preoperative WBC levels in patients with left-sided CRC can become a useful marker to rule out AL.

These results reflect the influence of tumor-related inflammation in patients with CRC on the healing process of the intestinal anastomosis created in their resections.

Generally, excessive inflammation is a key contributor to wound pathology, which lengthens recovery through the continued destruction of wound tissue. Along with elevated infiltration by specific immune cell subsets, pathological immune cell function is perturbed and collectively contributes to poor healing [27].

Buck et al. suggested that tumor necrosis factor-α, which is a proinflammatory cytokine predominantly produced by macrophages and tumor cells [28], can be a humoral mediator of impaired wound healing in patients with chronic diseases, including cancer associated with cachexia [29]. Additionally, tumors reportedly delayed wound closure in a murine animal model [30].

Taken together, tumors can potentially inhibit wound healing, even after being resected. Preoperative tumor-associated inflammation not only affects oncogenicity, but also reflect the patient’s ability to heal, including in intestinal anastomotic sites. However, the mechanisms and clinical effects are still unclear and should be studied in detail.

This study has some limitations. It is a single-institution, retrospective study. Nevertheless, as very few studies have focused on the relationships of preoperative inflammatory indicators and the occurrence of AL in left-sided CRC, the results presented here may help stratify patients who undergo surgery into high- and low-risk for AL.

To summarize, this study revealed that preoperative WBC count may help predict postoperative AL risk in left-sided CRC. It can make it possible to identify the risk of AL preoperatively and to take preventive methods efficiently. However, the predictive role of inflammatory indicators should be verified in larger-scale clinical studies. In particular, to use AL preventing methods more efficiently, it should be approached by interventional prospective studies with the preventing methods, stratified by preoperative WBC count.
